# Interdependencies of Gene Expression and Function between Two Redox Enzymes and REG Family Proteins in Murine Pancreatic Islets and Human Pancreatic Cells

**DOI:** 10.3390/antiox12040849

**Published:** 2023-04-01

**Authors:** Hong Wang, Marko Z. Vatamaniuk, Zeping Zhao, Xin Gen Lei

**Affiliations:** 1 Department of Animal Science, Cornell University, Ithaca, NY 14853, USA; 2 College of Light Industry and Food Technology, Zhongkai University of Agriculture and Engineering, Guangzhou 510225, China

**Keywords:** pancreatic islets, proliferation, redox, regeneration, viability

## Abstract

Our laboratory previously revealed that regenerating islets-derived protein 2 (REG2) was diminished in pancreatic islets of glutathione peroxidase-1-overexpressing mice (*Gpx1-OE*). It remained unknown if there is an inverse relationship between the expression and function of all *Reg* family genes and antioxidant enzymes in the pancreatic islets or human pancreatic cells. This research was to determine how altering the *Gpx1* and superoxide dismutase-1 (*Sod1*) genes alone or together (dKO) affected the expression of all seven murine *Reg* genes in murine pancreatic islets. In Experiment 1, *Gpx1^-/-^, Gpx1-OE*, their wild-type (WT), *Sod1*^-/-^, dKO, and their WT (male, 8-wk old, *n* = 4–6) were fed a Se-adequate diet and their islets were collected to assay the mRNA levels of *Reg* family genes. In Experiment 2, islets from the six groups of mice were treated with phosphate-buffered saline (PBS), REG2, or REG2 mutant protein (1 µg/mL), and/or GPX mimic (ebselen, 50 µM) and SOD mimic (copper [II] diisopropyl salicylate, CuDIPS, 10 µM) for 48 h before the proliferation assay using bromodeoxyuridine (BrdU). In Experiment 3, human pancreatic cells (PANC1) were treated with REG2 (1 µg/mL) and assayed for *REG* gene expression, GPX1 and SOD1 activities, viability, and responses to Ca^2+^. Compared with the WT, knockouts of *Gpx1* and/or *Sod1* up-regulated (*p* < 0.05) the mRNA levels of most of the murine Reg genes in islets whereas the *Gpx1* overexpression down-regulated (*p* < 0.05) Reg mRNA levels. REG2, but not the REG2 mutant, inhibited islet proliferation in *Gpx1* or *Sod1*-altered mice. Such inhibition was abolished by co-incubation the *Gpx1^-/-^* islets with ebselen and the *Sod1^-/-^* islets with CuDIPS. Treating PANC1 cells with murine REG2 protein induced expression of its human orthologue *REG1B* and three other *REG* genes, but decreased SOD1 and GPX1 activities and cell viability. In conclusion, our results revealed an interdependence of REG family gene expression and/or function on intracellular GPX1 and SOD1 activities in murine islets and human pancreatic cells.

## 1. Introduction

Reactive oxygen species (ROS) are involved in pathogeneses of chronic diseases including diabetes and cancer [1,2,3,4]. Se-dependent glutathione peroxidase 1 (GPX1) [5,6] and Cu-Zn superoxide dismutase (SOD1) [7] represent two major scavengers of intracellular ROS. Our laboratory has applied knockouts of GPX1 (*Gpx1^-/-^*) [8], SOD1 (*Sod1^-/-^*), [9] and both (dKO) [10,11] mice and GPX1-overexpressing (*Gpx1-OE*) mice [12,13] to study the metabolic roles and molecular mechanisms of these redox enzymes in the development of insulin resistance and diabetes. Paradoxically, overexpression of *Gpx1* in mice induced a type 2 diabetes-like phenotype, featuring augmented glucose-stimulated insulin secretion (GSIS) and chronic hyperinsulinemia [12,13]. Most strikingly, we found a diminished expression of regenerating islet-derived 2 (REG2) mRNA and protein in the *Gpx1-OE* islets [14]. A similar inverse relationship between GPX1 and REG2 expression was shown in high-fat diet-induced diabetic mice [15]. Subsequently, we revealed that the down-regulation of *Reg2* expression in the GPX1-overproducing pancreatic islets was mediated by a transcriptional inhibition of the gene through two ROS responsive transcription factors: activator protein-1 (AP-1) and albumin D box-binding protein (DBP) [14]. Because ROS-generating compounds diquat, streptozotocin, and hydrogen peroxide counteracted GPX1 overproduction on pancreatic islet REG2 protein and/or secretion, while ROS-scavenging compounds ebselen and N-acetylcysteine duplicated these effects, we proposed that ROS and GPX1 are counter-acting regulators of *Reg2* expression [14].

REG2 is one of the seven murine REG family proteins which include REG1, REG2, REG3α, REG3β, REG3γ, REG3δ, and REG4, and their respective genes are localized on chromosome 3 or 6 [16,17]. Because these REG proteins are abundantly produced in the pancreas and can be substantially induced by pancreatectomy [18], they are considered to be important for pancreatic regeneration [19,20]. They have also been shown to function as acute phase reactants with antiapoptotic and growth-promoting effects in islet β-cells, hepatocytes, and neuronal and epithelial cells [21,22,23] as well as in different tissues [24,25,26]. Because the excessive production of GPX1 activity in *Gpx1-OE* mice suppressed *Reg2* expression in the pancreatic islets [14], it becomes interesting to determine if the inverse relationship between *Reg2* gene expression and GPX1 activity could extend to all murine *Reg* family genes and alterations of another ROS-scavenging enzyme SOD1. Specifically, it is meaningful to find out if knockout of *Gpx1* induces responses of these *Reg* genes opposite to those in the *Gpx1-OE* mice and if knockouts of *Gpx1* and *Sod1* alone and together exert similar effects on *Reg* family gene expression. Functionally, it is important to find out if effects of exogenous REG2 on cell proliferation are modulated by altering *Gpx1* and/or *Sod1* expression as well as by the GPX mimic ebselen and SOD mimic CuDIPS.

In the present study, we isolated islets from the pancreases of *Gpx1^-/-^, Sod1^-/-^, Gpx1^-/-^Sod1^-/-^ (dKO*), and *Gpx1-OE* mice and their respective wild-type (WT) controls. We determined effects of altering these antioxidant enzyme genes on: (1) mRNA abundances of the seven murine *Reg* family genes in pancreatic islets and (2) cell proliferation responses of cultured islets to treatments with exogenous, recombinant murine REG2 protein. To explore the biomedical potential of recombinant REG proteins, we treated a human pancreatic cell line (PANC1) with murine REG2 protein and evaluated its effects on *REG* gene expression, cell viability, intracellular GPX and SOD1 activities, and its interactions with Ca^2+^.

## 2. Materials and Methods

### 2.1. Animals, Tissue Sample Collection, and Islet Isolation

Our animal protocols were approved by the Institutional Animal Care and Use Committee of Cornell University. Male mice (8 weeks of age) were housed in boxes at 20–26 °C and 40–60% humidity under a 12 h light/dark cycle. They had free access to food containing adequate levels of all required nutrients (0.3 mg Se/kg) and water. *Gpx1^-/-^*, *Sod1^-/-^*, and their WT littermate mice were generated from C57BL/6 mice [27,28]. The dKO mice were generated in our laboratory by crossing the *Gpx1^-/-^* and *Sod1^-/-^* knockouts [9]. The *Gpx1-OE* mice were derived from a B6C3 (C57B1 × C3H) hybrid line [8]. The genotypes of the mice were confirmed by PCR using tail genomic DNA as the template. After overnight food deprivation, the experimental mice (*n* = 4–6 per genotype) were euthanized by CO_2_ and pancreatic islets were isolated using a standard procedure with minor modifications [12].

### 2.2. In Vitro Islets Treatment and Proliferation Assay

The pancreatic islets (400 per sample, *n* = 6 mice per genotype) from all the six genotypes were cultured in RPMI 1640 (Gibco, Grand Island, NY, USA) containing 10% fetal bovine serum (FBS) and 1% antibiotic solution, and incubated in a humidified atmosphere of 5% CO_2_ at 37°C. The pancreatic islets were incubated with murine REG2 protein (2 μg/mL), murine REG2 mutant (2 μg/mL, Reg2 Mut), copper diisopropylsalicylate (CuDIPS, 10 μM) (Sigma-Aldrich, St. Louis, MO, USA), or ebselen (50 µM) (Sigma-Aldrich) for 48 h before the proliferation assay. The murine REG2 and REG2 Mut proteins were expressed in *Pichia pastoris* [29]. Briefly, the NOD mouse *Reg2* cDNA (NM 009043, G22 to A173) [24] was inserted into the *Pichia pastoris* expression vector pPICZ (Invitrogen, Grand Island, NY, USA). The mutant contained substitutions of three amino acid residues in the C-type lectin binding domain [30] with alanine (G142A, K157A, and D158A) and was created by site-directed mutagenesis of the *Reg2*/pPICZ gamma C construct through touchdown PCR (Integrated DNA Technologies, Coralville, IA, USA). Isolated *Reg2* plasmids were transformed into the yeast *P. pastoris* GS200 using electroporation (BTX, Holliston, MA, USA), and cultured in BMMY media for 5 d. The secreted REG2 proteins were isolated and purified by ammonium sulfate precipitation and ion-exchange chromatography, and verified by SDS-PAGE and immunoblotting.

The proliferation of pancreatic islets was determined using 10 μM bromodeoxyuridine (BrdU, Sigma-Aldrich), following the manufacturer’s instructions. After fixation with 70% ethanol, the cells were incubated with 1.5 M HCl for 30 min and washed with PBS. Thereafter, the islets were incubated with the BrdU antibody (Invitrogen) at room temperature for 1 h in the dark, and then washed with PBS. The islets were stained with the anti-mouse secondary antibody (Invitrogen) at room temperature in the dark for 1 h. After the cells were washed with PBS, nuclear counterstaining was performed using 4′,6-diamidino-2-phenylindole (DAPI, Invitrogen). Immunofluorescence images were acquired with a Zeiss LSM 710 confocal microscope (Carl Zeiss, Aalen, Germany) and evaluated using the Image J software version 1.53f 25 (https://imagej.net/ij/index.html; accessed on 2 November 2019).

### 2.3. Cell Culture and Viability Assay

Human pancreatic cancer cells, PANC1, were maintained in DMEM (Gibco, Grand Island, NY, USA) containing 10% FBS and 1% antibiotic solution. The cells were incubated in a humidified atmosphere of 5% CO_2_ at 37 °C. The incubation media was replaced every 48 h. Cell viability was assessed as previously described. Briefly, cells in growth media were plated in 24-well plates at 5 × 10^4^ cells/well and incubated at 37 °C in 5% CO_2_ for 24 h. Then, the medium was replaced by the growth medium and incubated for another 20 h. The number of viable cells was counted using a hemocytometer based on the trypan blue exclusion method [31].

### 2.4. Quantitative Real-Time PCR and Enzyme Activity Assays

Total RNA was isolated from freshly prepared islets (*n* = 6 mice/group, 150 islets per sample) and PANC1 cells using TRIzol reagent (Invitrogen) according to the manufacturer’s instructions. Reverse transcription of RNA and real-time PCR were carried out using one step SYBR Green Master Mix regents and an ABI 7700 (Applied Biosystems, Foster City, CA, USA). The normalized expression levels of the genes were calculated by the 2^−ΔΔCt^ method, using β-actin, a ubiquitous cytoskeletal protein, as a control gene. The primers used are listed in Table S1. SOD1 activity was measured using a water-soluble formazan dye kit (Dojindo Molecular Technologies, Gaithersburg, MD, USA) according to the manufacturer’s instructions. GPX activity was determined using the coupled assay of NADPH oxidation using H_2_O_2_ as the substrate [32].

### 2.5. Statistical Analysis

**Figure 1:**
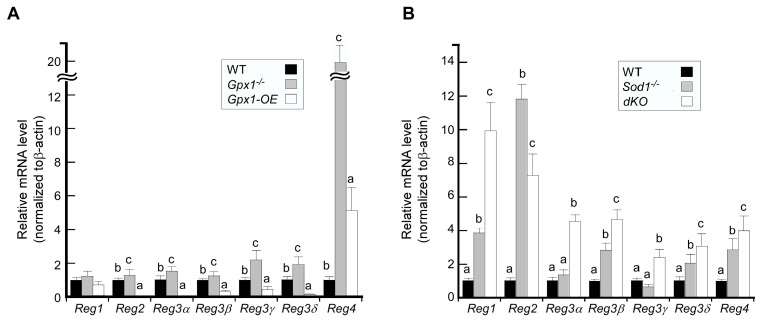
Relative abundance of *Reg* mRNA in pancreatic islets of wild-type (WT), *Gpx1^-/-^*, and *Gpx1-OE* mice (**A**), and WT, *Sod1^-/-^*, and *Gpx1^-/-^Sod1^-/-^* (*dKO*) mice (**B**). Gene expression was quantified by Q-PCR and normalized with β-actin expression. Values were expressed as relative expression levels to respective WT and presented as mean ± SD. Data were analyzed using one-way analysis of variance (ANOVA) for individual genes. Means of the same gene without sharing a common letter differ at *p* < 0.05; *n* = 4–6.

Data were presented as mean ± standard deviation (SD). The effects of genotypes (*Gpx1^-/-^* or *Gpx1-OE* vs. *WT* and *Sod1^-/-^* or *DKO* vs. *WT*) on the mRNA levels of each *Reg* family gene (Figure 1) were analyzed using one-way analysis of variance (ANOVA) for individual genes. Duncan’s multiple comparison post-test was used for the mean comparisons. Data from the rest of study were analyzed using Student’s *t* test to compare the various treatment effects with the respective controls. The statistical significance was set at *p* < 0.05. All statistical analyses were performed using SPSS 22.0 (SPSS Inc., Chicago, IL, USA).

## 3. Results

### 3.1. Effects of Knockouts of Gpx1 and/or Sod1 on Expression of Reg Family Genes in Mouse Islets

Compared with WT islets, *Gpx1^-/-^* islets had greater (*p* < 0.05) mRNA levels of all seven *Reg* genes except for *Reg1* (Figure 1A). The difference between these two genotypes in *Reg4* was approximately 20-fold (*p* < 0.05). In contrast, *Gpx1-OE* islets showed nearly diminished or substantially decreased (*p* < 0.05) mRNA levels of all *Reg* genes except for *Reg1* and *Reg4* compared with the WT islets. Intriguingly, the *Reg4* mRNA level of *Gpx1-OE* islets was approximately 5-fold higher (*p* < 0.05) than that of the WT islets.

As shown in Figure 1B, the mRNA levels of four *Reg* genes, but not *Reg2*, *Reg3*α, or *Reg3*γ, showed graded increases (*p* < 0.05) from the WT to *Sod1^-/-^* to dKO islets. In contrast, *Sod1^-/-^* islets had greater (*p* < 0.05) increases in *Reg2* mRNA levels than dKO islets, and only the dKO islets had greater (*p* < 0.05) mRNA levels of *Reg3*α and *Reg3*γ compared to the WT islets.

### 3.2. Impacts of GPX1 and SOD1 Status on Inhibitions of Islet Proliferation by Exogenous REG2 Protein

**Figure 2:**
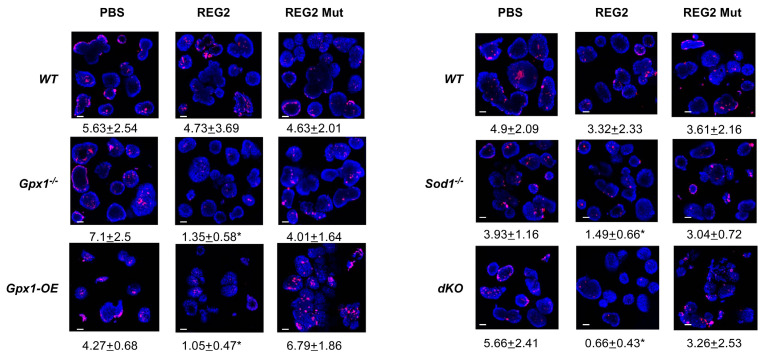
BrdU (red) immunostaining of pancreatic islets (blue) isolated from WT, *Gpx1^-/-^, Gpx1-OE, Sod1^-/-^*, and *dKO* mice. Islets were incubated with REG2 and REG2 Mut for 48 h before the proliferation assay. Numbers under the image panel represent relative BrdU fluorescent intensity, and means with * differ at *p* < 0.01 compared with the PBS control. Data were analyzed using Student’s *t* test within each genotype. Representative images are displayed *for* each condition; *n* = 4. Scale bar = 50 μm.

Compared with the respective PBS treatments (Figure 2), the incubation with REG2 protein led to substantial reductions of BrdU incorporation into islets of the four genetically altered groups of mice. Specifically, the staining intensity was decreased (*p* < 0.01) by 81% in *Gpx1^-/-^* islets, 75% in *Gpx1-OE* islets, 62% in *Sod1^-/-^* islets, and 88% in dKO islets. However, the incubation with the same amount of REG2 mutant (REG2 Mut) protein produced no significant changes in BrdU incorporation or staining intensity in islets of any of these four genotypes compared with the PBS treatments. The two groups of WT islets showed no response to the treatments with either REG2 or REG2 Mut compared with the PBS treatments.

**Figure 3:**
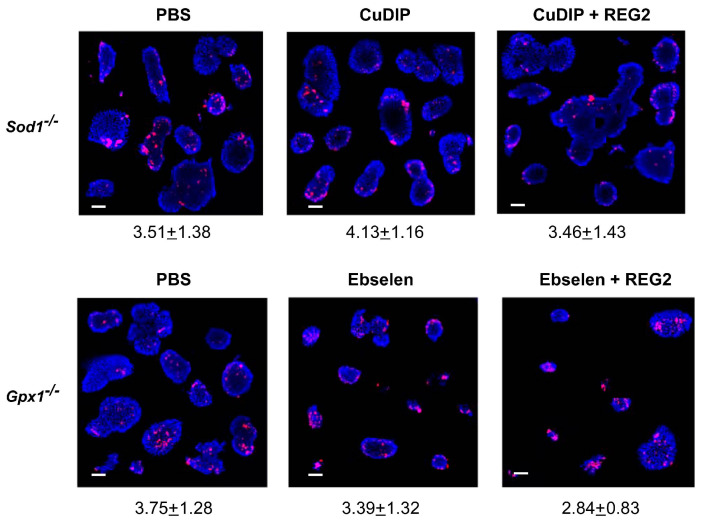
Effects of co-incubation with GPX mimic ebselen or SOD mimic CuDIP on the REG2-mediated inhibition of islet proliferation measured by the BrdU (red) immunostaining in the *Gpx1^-/-^* and *Sod1^-/-^* islets. Numbers under the image panel represent relative BrdU fluorescent intensity. Data were analyzed using Student’s *t* test within each genotype. Images are representative of replicated experiments: *n* = 3–4. Scale bar = 50 μm.

Meanwhile, incubating the *Gpx1^-/-^* islets with ebselen and the *Sod1^-/-^* islets with CuDIPS (Figure 3) did not produce apparent BrdU staining changes over their respective controls treated with PBS. Strikingly, the inhibition of BrdU incorporation by REG2 disappeared in the *Gpx1^-/-^* islets co-incubated with ebselen or in the *Sod1^-/-^* islets co-incubated with CuDIPS.

### 3.3. Effects of Murine REG2 Protein on REG Gene Expression and Antioxidant Enzyme Activities in PANC1 Cells

**Figure 4:**
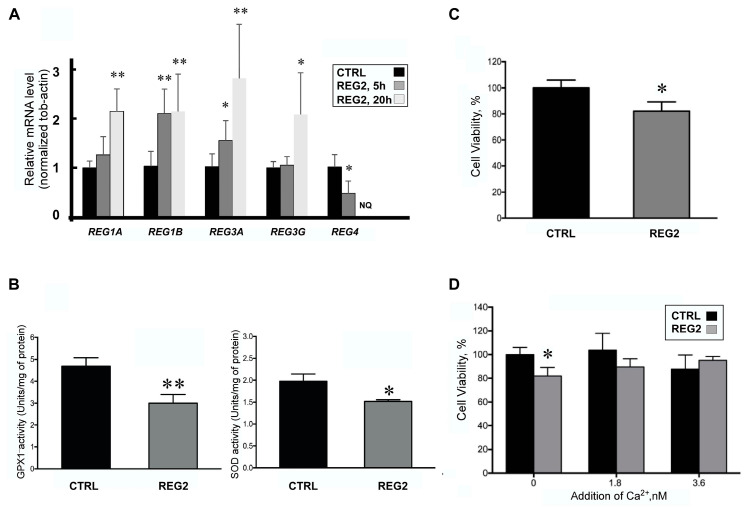
Effects of treating human PANC1 (cancer) cells with murine REG2 protein on mRNA levels of 5 human *REG* genes [(**A**), after 5 and 20 h of treatment], GPX1 and SOD activities [(**B**), after 20 h of treatment], cell viability [(**C**), after 20 h of treatment], and cell viability at three doses of additional calcium ions [(**D**), after 20 h of treatment]. Columns with * or ** differ significantly at *p* < 0.05 or *p* < 0.01 from control, respectively. Data were analyzed using Student’s *t* test within each REG gene (**A**) or calcium concentration (**D**).

To determine if murine REG2 exerted a cross-species effect on the expression of human *REG* genes, we treated PANC1 cells with recombinant murine REG2 for 5 or 20 h. The treatment elevated (*p* < 0.05 or 0.01) mRNA levels of *REG1A*, *REG1B*, *REG3A*, and *REG3G* compared with the controls. In contrast, the treatments decreased (*p* < 0.05) or diminished the expression of *REG4* (Figure 4A). Incubating PANC1 cells with the recombinant murine REG2 for 20 h decreased GPX (*p* < 0.01) and SOD1 (*p* < 0.05) activities by 20–30% compared with the control (Figure 4B).

### 3.4. Effects of Murine REG2 Protein on Cell Viability and Its Dependence on Free Calcium in PANC1 Cells

To explore putative metabolic roles of murine REG2 in human cell growth and survival, we tested the effects of the recombinant murine REG2 protein on the viability of PANC1 cells (Figure 4C). Indeed, the cell viability was decreased (*p* < 0.05) by the recombinant murine REG2 treatment, suggesting a cross-species effectiveness. Because we revealed a potential of REG2 as a novel regulator of calcium channel activity in islets [29], we tested if adding extra free calcium to the media could affect the decrease in viability of PANC1 cells caused by the REG2 treatment (Figure 4D). In fact, adding 1.8 or 3.6 nM of free Ca^2+^ into the media attenuated the REG2-mediated decrease in cell viability.

## 4. Discussion

Our present study revealed a largely inverse relationship between murine *Reg* family gene expression and intracellular ROS-scavenging GPX1 and SOD1 activities in the pancreatic islets, with several exceptions. While the *Gpx1^-/-^* and *Sod1^-/-^* islets had higher mRNA levels of most of the *Reg* genes than their respective WT islets, the *dKO* islets showed further elevations in or higher mRNA levels of six genes compared to the *Sod1^-/-^* islets. In contrast, the *Gpx1-OE* islets had lower or diminished expression of five genes, the exceptions being *Reg1* and *Reg4,* compared with the WT islets. This extends our earlier finding on the down-regulation of *Reg2* expression in the pancreatic islets of *Gpx1-OE* mice [14] to a rather common negative interaction in gene expression between the two major antioxidant enzymes GPX1 and SOD1 and *Reg* family genes. Alternatively, our previously illustrated counteracting regulations of *Reg2* by ROS and GPX1 [14] might be viewed as an interdependence of intracellular ROS-scavenging enzyme activity and *Reg* family gene expression [14]. Our findings may qualify antioxidant enzymes and their counterpart ROS as strong regulators of *Reg* family gene expression that is up-regulated by pancreatectomy [18] and pro- and anti-inflammatory cytokines [33,34,35]. It is well known that pancreatic islets have a low baseline level of antioxidant enzyme expression [36]. This feature allows islets to possess the high ROS sensitivity required for regulating insulin secretion [3]. Our results suggest that altering ROS production may influence islet functions through affecting the expression of *Reg* genes. It is puzzling that up-regulated *Reg* expression in the *Gpx1^-/-^*, *Sod1^-/-^*, and *dKO* islets was associated with decreased islet β-cell mass and insulin secretion [11], while down-regulated *Reg* expression in *Gpx1-OE* islets was accompanied with islet hyperplasia, hyperinsulinemia, and augmented glucose-stimulated insulin secretion [12,13].

Despite the demonstrated main trends, there were several intriguing exceptions for the relationship between antioxidant enzymes and *Reg* family gene expression in murine pancreatic islets. Although we showed that down-regulation of *Reg2* expression in the GPX1-overproducing pancreatic islets was mediated by a transcriptional inhibition of the gene through two ROS-responsive transcription factors AP-1 and DBP [14], we could not find a uniform presence of such domains in the proximate promoters of the rest of the *Reg* family genes. Thus, intracellular GPX1 and SOD1 likely regulate their transcription through other transcriptional factors or mechanisms. Compared with other *Reg* genes, *Reg1* expression was not altered whereas *Reg4* expression was much more substantially (~20 fold) elevated by the knockout of *Gpx1*. Meanwhile, the mRNA levels of islet *Reg3*α and *Reg3*γ were not affected by knockout of *Sod1* which induced greater levels of *Reg2* expression than the double knockouts. Seemingly, the regulation of *Reg* family gene expression by GPX1 and SOD1 alterations involves additional mechanisms associated with specific ROS species instead of just total levels, individual responsive elements in the *Reg* gene promoters, and their interactions. Most notably, islet *Reg4* expression was induced by both knockout and overproduction of GPX1. These unilateral responses to both increases and decreases in intracellular antioxidant enzyme activities were distinctly different from those of the other six *Reg* genes. Whereas a detailed mechanism for this difference remains to be revealed, *Reg4* is the most phylogenetically different gene within the *Reg* family. In mice, the other six *Reg* genes were mapped to a 75 kb region of chromosome 6C while *Reg4* is located on chromosome 3 [37]. In humans, four of the five *REG* genes are located in a 95 kb region of chromosome 2p12 [38], but *REG4* is on chromosome 1 [39].

Another interesting finding of our study is the inhibition of proliferation by exogenous REG2 protein in the *Gpx1^-/-^* and *Sod1^-/-^*, but not WT, islets. While REG2 Mut had no such effect on islet proliferation, GPX1 and SOD1 mimics obviated the REG2-mediated inhibition. This raises a serious question against the potential of REG2 as a crucial growth factor for pancreatic islet proliferation [40]. In fact, *Reg2^-/-^* mice maintained normal β-cell mass until later stages of life or the onset of obesity induced by a high-fat diet [25]. As mentioned above, we have shown that the diminished *Reg2* expression in the pancreatic islets of *Gpx1-OE* mice was associated with elevated β-cell mass [12]. In contrast, pancreatic β-cell mass was decreased in the *Gpx1^-/-^* and *Sod1^-/-^* mice [12] that expressed greater levels of *Reg* genes than the WT mice. Reciprocally, a pancreatic acinar overexpression of *Reg2* did not confer any protection against pancreatitis or diabetes [41]. Seemingly, we should not simply view REG2 as a “default” growth factor for intact or unstressed islet β-cells or other types of cells. Instead, it may function as an acute stress reactant in regulating the growth or survival of cells with altered or compromised metabolism. Thus, our study suggests a link between the function and the outcome of REG2 to the intracellular redox status or antioxidant activity context. This may help explain why *exogenous* REG2 inhibited the islet proliferation of *Gpx1^-/-^* and *Sod1^-/-^* mice but not the WT mice, why REG2 inhibited islet proliferation in both *Gpx1*^-/-^ and *Gpx1-OE* mice, and why restoring normal redox status with GPX1 and SOD1 mimics precluded such inhibition. In fact, REG2 was shown to inhibit nuclear entry of apoptosis-inducing factor by promoting the nuclear presence of Scythe and inducing heat shock protein 70 (HSP70) in mouse insulinoma cells [42]. In addition, *Gpx1* overexpression was associated with a thermo-sensitive phenotype via a direct participation of peroxides in the induction of cytoprotective protein HSP70 [43]. A possible extended linking of GPX1-HSP70-Scythe-REG2 may depict a dependence of cell apoptosis/division on its redox state [44]. In addition, REG3β is considered unlikely to be an islet growth factor but a putative protector that prevents streptozotocin-induced damage by inducing expression of specific genes [45]. Expression of human REG1B, an orthologue of mouse REG2, is altered under conditions of inflammatory diseases [33,34,35], cancer [46], and childhood stunting [33].

Our third interesting finding is that exogenous murine REG2 protein decreased the viability of human pancreatic cancer cells (PANC1), and the inhibition disappeared after the addition of extra free Ca^2+^ ions into the media. This action was consistent with its inhibition of the proliferation of *Gpx1^-/-^* and *Sod1^-/-^* islets, along with a down-regulation of both GPX and SOD activities in the cells. These results suggest a cross-species effectiveness of REG2 with an anti-human cancer potential and an involvement of calcium channels in the mechanism [33,46]. The lack of inhibition of islet proliferation by REG2 Mut (with a destruction of the C-type lectin binding domain) supports this notion. Interestingly, murine REG2 enhanced the expression of *REG1A*, *REG1B*, *REG3A*, and *REG3G,* but decreased or diminished the expression of *REG4* in PANC1 cells. Different types of cancer are associated with increased level of REG family proteins [47]. REG1A, REG1B, and REG3A were reported to be increased in cancer ductal fluid [48], and REG1A was linked to pancreatic cancer [47]. However, the metabolic functions of REG3β remain controversial. Gironello reported that REG3β deletion in mice drastically impaired pancreatic tumor growth, correlating with decreased angiogenesis and increased apoptosis of tumor cells [48]. Liu and colleagues reported that REG3β negatively regulated cytokine-induced activation of STAT-3 in colon epithelial cells thus reducing carcinogenesis [49]. However, in HIP/PAP (REG3α) knockout mice, PAP showed an antiapoptotic and anti-inflammatory effect during cerulean-induced acute pancreatitis [50], while antisense knockdown of PAP expression exacerbated the severity of pancreatitis, suggesting a protective function against pancreatitis [51].

## 5. Conclusions

Altering GPX1 and SOD1 activities, either by single or double knockout of the two major ROS-scavenging enzymes, enhanced the expression of most murine *Reg* family genes in the pancreatic islets. These increases were contrary to the down-regulation induced by the overproduction of GPX1 activity in the islets. However, there were variations with individual *Reg* genes and antioxidant enzymes. Exogenous murine REG2 protein inhibited the proliferation of *Gpx1^-/-^* and *Sod1*^-/-^ islets and decreased cell viability of human pancreatic cancer cells (PANC1). Meanwhile, these actions of REG2 were obviated by the additions of GPX and SOD mimics or free Ca^2+^ ions, and thus there was no simple cascade to explain the underlying mechanism. Although additional research is still needed to completely unveil the complexity of this matter, our current findings reveal an interdependence of gene expression and function between two redox enzymes and the REG family proteins in murine pancreatic islets and human pancreatic cancer cells.

## Data Availability

All data are contained within the article.

## Supplementary Materials

The following are available online at https://www.mdpi.com/article/10.3390/antiox12040849/s1, Table S1: Primers used for Q-PCR analysis.
