# Supplementary material for: Interdependencies of Gene Expression and Function between Two Redox Enzymes and REG Family Proteins in Murine Pancreatic Islets and Human Pancreatic Cells
Source: Antioxidants (Basel). 2023 Apr 1;12(4):849. doi: 10.3390/antiox12040849 (PMC10135238; doi:10.3390/antiox12040849)
Supplement: Supplementary file 1 [file antioxidants-12-00849-s001.zip › antioxidants-2201446-supplementary.pdf]

Supplemental Table S1. Primers used for Q-PCR analysis

Gene Name	Accession number	Product size	Primer (5`-3`)	
Murine genes				
β-actin	NM_007393.5	156	Forward	TCATGAAGTGTGACGTTGACATC
			Reverse	CAGGAGGAGCAATGATCTTGATCT
Reg1	NM_009042.2	111	Forward	GACTCCATGATCCCAAAAGG
			Reverse	CACAGTAGCCACGATTGGAA
Reg2	NM_009043.2	107	Forward	TCCCCTTGGCTGAAAAAGAC
			Reverse	CCAGGTCAAACGGTCTTCAA
Reg3α	NM_011259.1	107	Forward	AGGCTTCCTTTGTGTCCTCCTT
			Reverse	TCCACCTCCATTGGGTTGTTGA
Reg3β	NM_011036.1	175	Forward	AAGACAGACAAGATGCTGCCTCCA
			Reverse	ACAAGGCATAGCAGTAGGAGCCAT
Reg3γ	NM_011260.2	149	Forward	TCCTTTCTCAGGTGCAAGGTGA
			Reverse	TTGGCAGGCCATATCTGCATCA
Reg3δ	NM_013893.2	173	Forward	AGCATGTCCTGGATGCTACTGTGT
			Reverse	ATCTCTGCATTAGCCCAGGTCTGT
Reg4	NM_026328.2	123	Forward	ATCAGAGAAACCTGCCTGTGTGGA
			Reverse	TGGCTTCACTCTTTGTCCTGGGAT
Human genes				
β-ACTIN	NM_001101.5	184	Forward	AGAGCTACGAGCTGCCTGAC
			Reverse	AGCACTGTGTTGGCGTACAG
REG1A	NM_002909.5	195	Forward	CCTTTGTGGCCTCACTGATT
			Reverse	GGAATCCTGTGCTTGAGGTC
REG1B	NM_006507.4	164	Forward	CCAACTCGTTCTTCATGCTG
			Reverse	AACCCAGGTCTCAGGGTCTT
REG3A	NM_138937.3	93	Forward	AGAGAATATTCGCTTAATTCC
			Reverse	AATGAAGAGACTGAAATGACA
REG3G	NM_001008387.3	90	Forward	GAATATTCTCCCCAAACTG
			Reverse	GAGAAAAGCCTGAAATGAAG
REG4	NM_001159352.2	86	Forward	ATCCTGGTCTGGCAAGTC
			Reverse	CGTTGCTGCTCCAAGTTA

Abbreviation: Reg/REG: regenerating islet-derived protein.
